# Aerosol-based ligand soaking of reservoir-free protein crystals

**DOI:** 10.1107/S1600576721003551

**Published:** 2021-05-28

**Authors:** Breyan Ross, Stephan Krapp, Ruth Geiss-Friedlander, Walter Littmann, Robert Huber, Reiner Kiefersauer

**Affiliations:** a Max Planck Institut für Biochemie, D-82152 Martinsried, Germany; b Proteros Biostructures GmbH, D-82152 Martinsried, Germany; c Center of Biochemistry and Molecular Cell Research, Albert-Ludwigs-Universität, D-79104 Freiburg, Germany; d ATHENA Technologie Beratung GmbH, Technologiepark 13, D-33100 Paderborn, Germany; e Zentrum für Medizinische Biotechnologie, Universität Duisburg-Essen, D-45147 Essen, Germany; f Fakultät für Chemie, Technische Universität München, D-85747 Garching, Germany

**Keywords:** aerosols, soaking, ultrasonic, DPP8

## Abstract

Soaking of macromolecular crystals allows the formation of complexes via diffusion of molecules into a preformed crystal for structural analysis. Soaking offers various advantages over co-crystallization, *e.g.* small samples and high-throughput experimentation. However, this method has disadvantages, such as inducing mechanical stress on crystals and reduced success rate caused by low affinity/solubility of the ligand. To bypass these issues, the Picodropper was previously developed in the authors’ laboratory. This technique aimed to deliver small volumes of compound solution in response to crystal dehydration supported by the Free Mounting System humidity control or by IR-laser-induced protein crystal transformation. Herein, a new related soaking development, the Aerosol-Generator, is introduced. This device delivers compounds onto the solution-free surface of protein crystals using an ultrasonic technique. The produced aerosol stream enables an easier and more accurate control of solution volumes, reduced crystal handling, and crystal-size-independent soaking. The Aerosol-Generator has been used to produce complexes of DPP8 crystals, where otherwise regular soaking did not achieve complex formation. These results demonstrate the potential of this device in challenging ligand-binding scenarios and contribute to further understanding of DPP8 inhibitor binding.

## Abstract 2

A novel aerosol device for protein crystal complexation using ultrasonic vibrations is described.

## 1. Introduction   

Crystallographic determination of macromolecules in complex with small molecules is typically performed either by co-crystallization or by soaking methods. In soaking, small molecules diffuse into preformed macromolecular crystals where they bind to specific sites of the protein depending on the concentration and solubility of solute in the solvent, and on the temperature (Muller *et al.*, 2009 ▸). As a rule of thumb, an increment of 20°C doubles the solubility of organic molecules (Black & Muller, 2010 ▸). Moreover, the ratio between solubility and affinity is crucial for successful binding (Müller, 2017 ▸). Soaking is important because it allows the screening of various small molecules without the need for crystallization screenings, thus saving time and resources. However, solubility studies of small-molecule libraries of thousands of compounds have shown that the solubility of most organic molecules is below the desired range, and a high percentage do not dissolve at all (Guha *et al.*, 2011 ▸; Bergström *et al.*, 2007 ▸). Most importantly, soaking is often aggressive, can affect diffraction quality irreversibly and can even dissolve protein crystals. It is therefore desirable to have a technique with the potential of soaking single protein crystals in a gentle way using weak binders or rather insoluble compounds.

The Free Mounting System (FMS) is a humidity control instrument designed to bath protein crystals in an air stream to control humidity and crystal temperature for post-growth crystal treatment (Kiefersauer *et al.*, 2002 ▸). Similar systems are being used at synchrotron beamlines (Bowler *et al.*, 2015 ▸). An alternative method based on IR radiation has also been developed (Kiefersauer *et al.*, 2014 ▸). The FMS was complemented with a Picodropper (PD) device, which supplies solutions in the form of picolitre drops to the reservoir-free crystal surface. Crystals can be kept in a controlled environment, both by optically monitoring the macroscopic crystal change and by X-ray diffraction measurement of crystal order while soaking compounds (Böttcher *et al.*, 2011 ▸). However, the PD has disadvantages and limitations, among which are induced mechanical stress on crystals caused by shooting with single droplets and nozzle blockage of the PD induced by the precipitated ligand.

Here we present the development and application of the Aerosol-Generator (AeGe), a new gentle soaking device that complements the humidity control and uses ultrasonic vibration to generate aerosols of ligands to soak reservoir-free protein crystals. We tested the AeGe on dipeptidyl peptidase (DPP8) crystals using two ligands which evaded complex formation by regular soaking methods (Ross *et al.*, 2018 ▸). Both 1G244 (Jiaang *et al.*, 2005 ▸) and the E67-interacting loop (EIL) peptide (SLRFLFEGQRIADNH) (Pilla *et al.*, 2013 ▸) are inhibitors of DPP8 and DPP9. However, they are examples of water-insoluble and low-affinity compounds, respectively. Finally, the AeGe technique allowed us to successfully achieve complex formation of 1G244 and the EIL peptide with DPP8 crystals. We propose the AeGe as an alternative method in challenging soaking scenarios using single protein crystals.

## 2. Instrumentation, materials and methods   

### 2.1. Operation of the AeGe   

**Figure 1:**
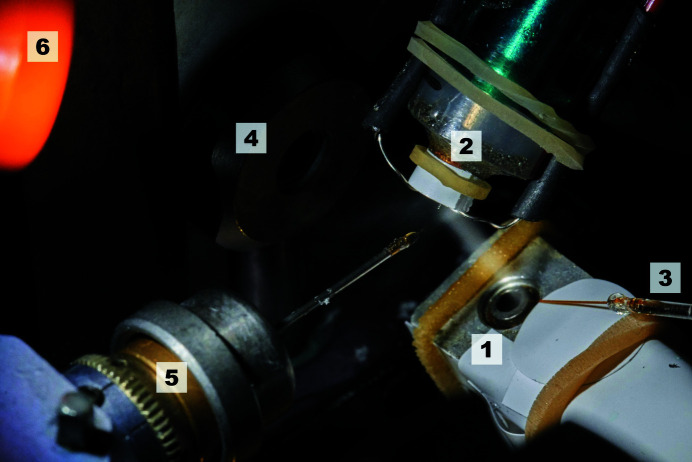
AeGe experimental setup. Shown is the AeGe (1) with the humidity nozzle (2), liquid supply capillary (3), collimator block (4), goniometer head with loop (5) and aerosol suction line (6).

The AeGe is an ultrasonic vibrating device designed to produce an aerosol from a liquid reservoir (Athena Company, Paderborn, Germany; https://www.myathena.de/). The aerosol is transported by the humid air stream from the humidity nozzle towards the protein crystal (Ellson *et al.*, 2003 ▸). Only a fraction of the aerosol (5%) reaches the probe, and thus the remaining aerosol is removed using air suction (Fig. 1 ▸).

The electronics connected to the AeGe induce a sinusoidal voltage signal of 250 kHz at the electrical terminals of the piezoelectric elements, setting the compound oscillator to vibrate in its first longitudinal resonance. The top ring area of the AeGe is the active surface, which is loaded with small volumes of solution that are vaporized as aerosol upon vibration via the acoustical capillary effect (Dezhkunov & Prokhorenko, 1980 ▸). The average drop size (*D*) of the aerosol depends on the ultrasonic frequency (*f*) according to (approximately) *D* ∼ (1/*f*)^2/3^. For water and *f* = 250 kHz, *D* is in the region of 8 µm (Reimann & Pohlman, 1976 ▸). The ultrasound generator simultaneously controls the sine wave amplitude and frequency to get a stable speed of the active surface, always tracking the resonance state using a phase-locked loop concept. The ultrasound generator is connected to a host computer via Ethernet, and thus the user can set a desired amplitude of the electrical signal. The ultrasonic vibration generates heat and mechanical stress inside the compound oscillator; therefore, oscillations and surface temperature are monitored by an oscilloscope and a thermocouple sensor, respectively. Moreover, to avoid overheating, a security shut-off system was implemented.

### 2.2. AeGe liquid supply, cooling and alignment   

**Figure 2:**
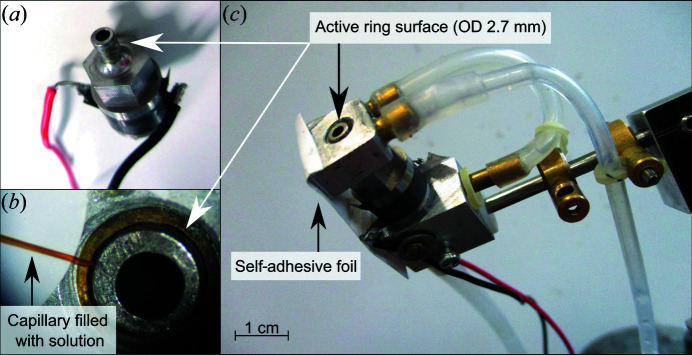
The AeGe. (*a*) Core unit of the AeGe. (*b*) Magnification of the active surface with the liquid supply capillary. (*c*) The AeGe core unit with cooling parts on top and bottom connected to the tubing for liquid cooling, the electrical wires, and means for co-axial alignment of the AeGe. The parts are held together by a self-adhesive foil fixing the AeGe.

The precise and gentle treatment of crystals with an aerosol solution is directly linked to the accurate supply of liquid to the AeGe. Solutions are delivered to the active surface of the AeGe via a thin flexible capillary [World Precision Instruments, microfilm products, inner diameter 100 µm, outer diameter (OD) 164 µm, length 10 mm]. Air pulses are software controlled between 5 and 20 ms using an on/off valve (LEE Hydraulische Miniaturkomponenten GmbH) which is connected to the capillary (Figs. 2 ▸ and 3 ▸). The air pulse length and the repetition rate control the amount of solution delivered to the AeGe over time, which is typically in the region of 10 nl s^−1^. The capillary is placed accurately by a mechanical micromanipulator to avoid direct contact with the active surface. Additionally, to prevent a continuous flow of liquid driven by the ultrasound when the liquid contacts the vibrating part, vacuum is applied to the capillary during the off-time of the valve. Thus, the liquid remains in the capillary and is no longer in contact with the AeGe. The capillary is filled by dipping it in the solution and applying vacuum. The same procedure is used to clean the capillary with water.

The AeGe was designed so as to minimize intrinsic mechanical friction. Nevertheless, for continuous operation, the AeGe has to be actively cooled to avoid overheating (Figs. 2 ▸ and 3 ▸). The working temperature of the active surface has to be controlled between 20 and 30°C to ensure stable and reproducible aerosol generation. At colder temperatures, the viscosity of liquids can be too high and aerosol formation is prevented, especially for the 100% dimethyl sulfoxide (DMSO) solution used in this study. Conversely, at higher temperature, salt solutions tend to crystallize, which hinders aerosol formation. The AeGe is positioned relative to the humid air stream by a holder, allowing rotations around three axes and translations in two orthogonal directions. The point to which the capillary is directed on the active ring area determines the spatial distribution of the aerosol and should not vary to ensure reproducibility.

### 2.3. Determination of crystal native humidity and solvent exchange   

The method described here is based on the exchange of bulk water of the crystal with the solvent (Böttcher *et al.*, 2011 ▸). The amount of bulk water replaced by the solvent is a function of the relative humidity (r.h.) of the humidified gas stream bathing the crystal. The quantitative relationship between relative humidity and solvent concentration inside a crystal can be obtained by comparing the 2D shadow projections (crystal area) collected at the native humidity of the crystal against the same crystal dehydrated by 20%, both at fixed crystal orientation. The difference in shadow area reflects the volume occupied by the solvent at the end of the humidity gradient. This step is performed in one test crystal before starting the soaking experiment. Here we found that the 20% shrinkage was often achieved with approximately 78% r.h. gas stream, which was then chosen as the target dehydration humidity for soaking with the AeGe in subsequent experiments.

### 2.4. Protein purification, crystallization and structure solution   

Protein purification, crystallization and structure solution were performed as described by Ross *et al.* (2018 ▸). Briefly, human cDNA of DPP8 isoform 1 (Uni-ProtKB Q6V1X1) was obtained from GeneArt. DPP8 (1898) protein was expressed in *Spodoptera frugiperda* cells (Sf9) and purified. Crystallization was performed by the hanging-drop method. DPP8 crystals grew at 4°C in 1:1 ratio drops of 10 mg ml^−1^ protein and 0.46 *M* Na citrate pH 6.75 as precipitant solution. Crystals of space group *C*222_1_ were soaked either by the classical powder soaking method or by the AeGe method. Soaking of 1 *M* tri­methyl­amine *N*-oxide (TMAO) was used as cryo-protectant. Data sets were collected at the SLS-X06SA beamline. Data sets were processed with *XDS* (Kabsch, 2010 ▸). The unbound DPP8 structure [Protein Data Bank (PDB; Berman *et al.*, 2000 ▸) entry 6eoo; Ross *et al.*, 2018 ▸] was used for molecular replacement using *Phaser* (McCoy *et al.*, 2007 ▸). The model was refined using restraints in *Refmac5* (Murshudov *et al.*, 1997 ▸). Coordinates and structure factors were deposited in the PDB with the following access codes: DPP8–SLRFLFEGQRIADNH PBD code 6trw and DPP8–1G244 PDB code 6trx. A summary of data collection and refinement statistics for each structure is provided in Table 1 ▸.

### 2.5. Determination of 1G244 solubility   

The solubility dependence of 1G244 on DMSO concentration was determined by measuring its absorbance peak at 262 nm relative to a 100% DMSO dilution standard curve (NanoDrop 1000, Thermo Fisher). 1G244 samples were diluted in water and incubated at 40°C for 24 h at 300 r min^−1^ shaking. Then, samples were spun down for 15 min at 20 000 *g*. Afterwards, samples were filtrated using a polyvinylidene fluoride filter of 0.22 µm (Millex-GV). The absorbance of the transparent eluted solution was measured at 25°C (S. Dalziel & K. Phizackerley, APPLICATION NOTE: NanoDrop 1000, *Small Molecule Crystallography*, Thermo Fisher Scientific, Wilmington, Delaware, USA). The absorbance of 1G244 at 25% DMSO was just above the assay noise signal. Thus, estimation of the saturation concentration at lower DMSO% was obtained by interpolation.

## 3. Results   

### 3.1. 1G244 solubility and DPP8 classical soaks   

**Figure 4:**
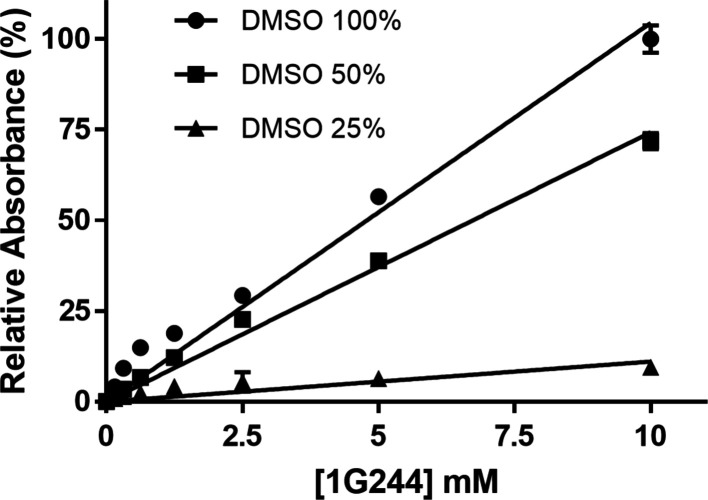
Water solubility of 1G244. Different concentrations of 1G244 were dissolved in decreasing concentrations of DMSO to determine the solubility of the ligand in water. 50% (squares) and 25% (triangles) DMSO dilutions were compared with a standard curve of 100% DMSO (circles). The absorbance of 1G244 was measured at 262 nm. Measurements were performed in triplicates and the absorbance of 10 m*M* 1G244 in 100% DMSO was used to normalize the measurements.

Even though 1G244 has Ki values of 0.9 and 4.2 n*M* for DPP8 and DPP9, respectively (Wu *et al.*, 2009 ▸), the failure to produce a DPP8 co-crystal with 1G244 raised the question of whether this inhibitor was available in sufficient amounts to form a complex in solution. Given that the hydro­phobic 1G244 small molecule precipitates after dissolution from a DMSO stock, its effective concentration was unknown. Therefore, we quantified the soluble fraction of 1G244 at low DMSO concentration using a NanoDrop-based technique. We started by preparing soluble 1G244 reference solutions (Fig. 4 ▸, 100% DMSO). The measured saturation concentration of 1G244 at 50 and 25% DMSO reached 75 and 10% of the initially calculated inhibitor concentration, respectively (Fig. 4 ▸). For 1–2% DMSO, the measured values of soluble 1G244 were below the sensitivity range of the NanoDrop. However, interpolation suggests that they are in the low nanomolar range, insufficient for complex formation and requiring a new method to increase the concentration of ligands.

**Figure 5:**
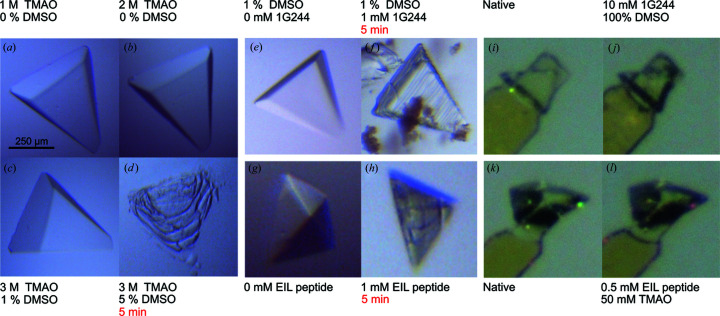
DPP8 crystal stability in different soaking solutions. (*a*)–(*d*) A DPP8 crystal is transferred sequentially to new drops with increasing concentrations of TMAO and DMSO, reaching up to 3 *M* and 5%, respectively. (*e*), (*f*) Classical soaking of a DPP8 crystal at 4°C. 1G244 was dissolved to 1 m*M* from a stock solution of 100 m*M* (precipitated 1G244 was readily visible). After 5 min the crystal cracked and then dissolved. (*g*), (*h*) Classical soaking of DPP8 crystals with 1 m*M* EIL peptide at 20°C. After 5 min the crystal cracked and then dissolved. All conditions had 0.46 *M* Na citrate as a base component. (*i*)–(*l*) DPP8 crystal mounted in the humidified air stream, before (native) and after spraying of 1G244 (*j*) and the EIL peptide (*l*) with the aerosol composition noted accordingly. In contrast to the classical soak experiments the crystals showed no visible degradation. Crystals showed long-term stability if time is not mentioned.

Apart from solubility, classical soaking experiments on DPP8 crystals with 1G244 are limited by crystal fragility. To understand what caused crystals to disintegrate, we transferred them to different solutions (Fig. 5 ▸). Crystals tolerated TMAO cryoprotectant, but increasing the DMSO concentration above 1% caused immediate crystal dissolution [Fig. 5 ▸(*a*)–5 ▸(*d*)]. Interestingly, DPP8 crystals in the reservoir were sensitive to 1G244 and the EIL peptide themselves, dissolving very quickly at 1 m*M* soaking concentration [Fig. 5 ▸(*e*) and 5 ▸(*f*)]. Conversely, soaking of either 1G244 or the EIL peptide was well tolerated under the FMS gas stream using the AeGe [Fig. 5 ▸(*i*)–5 ▸(*l*)].

### 3.2. Experiments with the AeGe: the soaking process   

**Figure 6:**
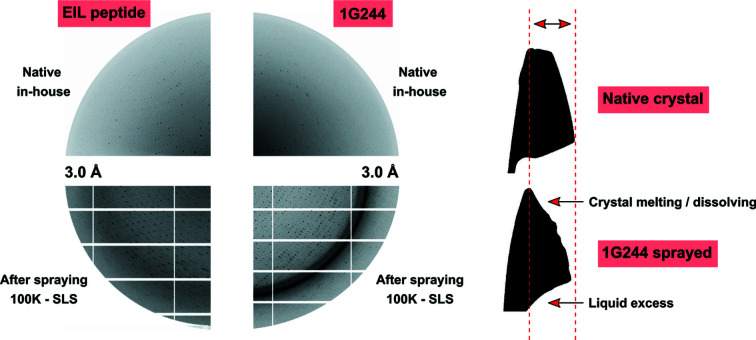
AeGe DPP8 treatments. Left: X-ray diffraction comparison of DPP8 native state, in-house X-ray source data (top), and after spraying, vitrified state, synchrotron data (bottom). Right: binary projections of a DPP8 crystal treated with 1G244 inhibitor, same orientation. The change in crystal shape before and after spraying indicates crystal instability and melting due to the detrimental effect of DMSO and 1G244 on the crystal structure. The dashed lines highlight that the crystal did not shrink/melt in the horizontal direction. For each ligand 10–15 crystals were used for method development and setting starting conditions (*e.g.* humidity, solvents, dehydration range). Five crystals were treated and sent to the synchrotron, yielding diffraction data up to 3–4 Å resolution (75% soaking success).

To avoid crystal fragility after 1G244 or DMSO treatments, solubility issues, and low binding affinity of EIL, the AeGe was used to perform soaking of DPP8 reservoir-free crystals with these ligands. A DPP8 crystal was transferred from the 4°C crystallization drop using a 4°C pre-cooled cryo-tong and mounted in the humidified gas stream (gas temperature 12.5°C, r.h. 97%). Then, the excess solution was removed and the native crystal quality was tested by X-rays (Fig. 6 ▸).

A pre-filled capillary with 10 m*M* 1G244 in 100% DMSO was positioned near the active surface of the AeGe. Simultaneously, an r.h. gradient was started from 97% down to 78% in 0.1% steps/12 s (35 min in total). The lost water inside the crystal resulting from the r.h. gradient was replaced by the aerosol solution, triggered at predefined area thresholds (2D shadow projection measurements; Böttcher *et al.*, 2011 ▸). As the humidity decreased, both ligand and DMSO were concentrated in the crystal. The final concentration of 1G244 was determined by the spraying time, whereas the final DMSO concentration was determined by the final humidity, which was sufficient for cryo-protection as well. Given that DMSO is moderately volatile, a continuous liquid supply was necessary to keep the crystal volume constant over time, thus ensuring crystal quality. After the end of the r.h. gradient (78%) the crystal was further sprayed with solution for 20 min. Then, the crystal became unstable, losing its shape, as observed by crystal projections (Fig. 6 ▸). Finally, the crystal was flash-cooled using the CryoSwitch (Kiefersauer *et al.*, 2014 ▸). The rings in the diffraction image are located at resolutions that match with ice rings (3.66 and 2.25 Å). Similarly, to soak 0.5 m*M* EIL peptide in a solution of 50 m*M* TMAO, an r.h. gradient was started at 97.3% with 0.1% steps/45 s down to 80% (2 h 10 min in total) with a gas temperature of 19°C. In contrast to the method employed to soak 1G244 (DMSO soluble), spraying the EIL peptide (water soluble) implies reaching an equilibrium at the end humidity, not crossing the lower optical shrinkage threshold, and thus the dehumidification stops automatically. Given that 4.5 *M* TMAO is approximately in equilibrium with 80% r.h., the equivalent accumulation of the EIL peptide in this experimental setup was theoretically 90-fold, resulting in 45 m*M* peptide as the final concentration in the crystal. Compared with the 3.7 m*M* active site concentration in *C*222_1_ DPP8 crystals, the excess of peptide in the crystal was beyond tenfold. TMAO serves both as cryoprotectant and as structure stabilizer and is regularly used in FMS applications. During the spraying process the crystal also melted to a certain degree, but it was later stable in contrast to the 1G244 DMSO treatment. Finally, the crystal was flash-cooled. Crystals lost diffraction power after soaking treatments, although it was sufficient to obtain data sets at the synchrotron.

### 3.3. Crystallographic results   

**Figure 7:**
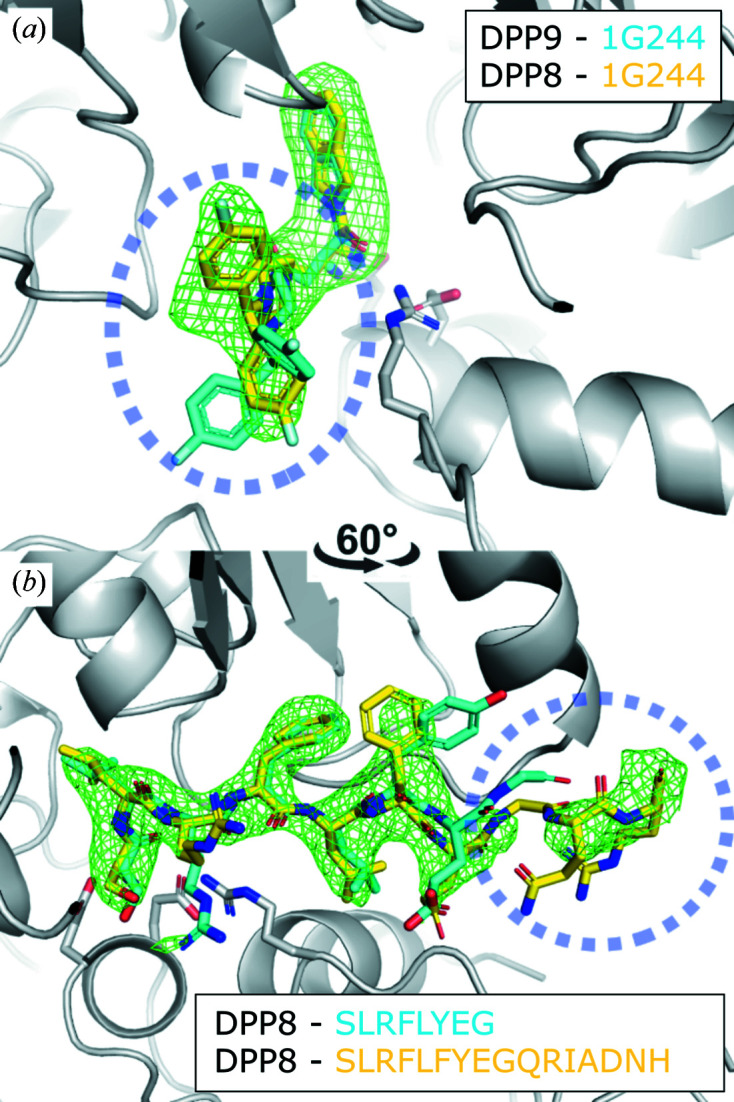
Crystallographic structures of DPP8 crystals soaked by AeGe. (*a*) Omit map of 1G244 (*F*_o_–*F*_c_, 3σ) soaked into a DPP8 crystal using the FMS–AeGe soaking method at 12.5°C. (*b*) Omit map of the EIL peptide (*F*_o_–*F*_c_, 3σ) soaked into a DPP8 crystal using the FMS–AeGe soaking method at 19°C. 1G244 and SLRFLYEG from 6eor and 6eop are, respectively, superposed for comparison (cyan; Ross *et al.*, 2018 ▸).

Using the AeGe we soaked DPP8 crystals with 1G244 and the EIL peptide, allowing us to solve both crystal structures (Table 1 ▸). The soaking procedure did not induce changes in the crystals, which is reflected in a conserved overall molecular structure when comparing the AeGe-soaked structures with the published DPP8 unbound structure 6eoo (0.34 Å Cα RMSD; Ross *et al.*, 2018 ▸). The crystallographic structure of the AeGe-soaked DPP8–1G244 crystals was determined at 3.2 Å. We identified positive electron density in the active site corresponding to 1G244 [Fig. 7 ▸(*a*)].

Unlike DPP9–1G244 (6eor; Ross *et al.*, 2018 ▸), the electron density is complete and well defined for all 1G244 atoms, at least in one of three protomers of the asymmetric unit. Similarly, we determined the crystallographic structure of the AeGe-soaked DPP8–SLRFLFEGQRIADNH crystals at 3.0 Å. Strong positive electron density is observed in the active site assigned to the EIL peptide, albeit not complete. We observed additional interpretable electron density for two residues with respect to the DPP8–SLRFLYEG crystal structure (6eop; Ross *et al.*, 2018 ▸) [Fig. 7 ▸(*b*)]. Interestingly, the binding mode of the EIL peptide closely resembles that of SLRLFLYEG, first in the anchoring of its *N*-terminus to two glutamic acids, and second by the induced-fit formation of an S2′ hydro­phobic sub-site to allocate the F4 residue. Moreover, both ligands induced the ordering of the R segment, a common feature of peptide-like binders.

To demonstrate that this method is applicable to other crystal systems, we aerosol-soaked cycline-dependent kinase 2 (cdk2) crystals with the specific ligand staurosporine using the AeGe. In response, we observed a strong positive electron density in the active site of the kinase, which confirmed complex formation (PDB code 7nvq; Supplementary Fig. 1).

## 4. Discussion   

Recent crystal soaking developments have directed attention toward high-throughput methods, thus meeting the need of the pharmaceutical industry for enabling fast-track drug discovery (Jiaang *et al.*, 2005 ▸; Collins *et al.*, 2018 ▸; Lieske *et al.*, 2019 ▸). Moreover, ultrasound-based soaking methods have already been developed to dispense compounds directly into crystallization drops (Ellson *et al.*, 2003 ▸; Collins *et al.*, 2017 ▸). However, these soaking approaches are not ideal in cases where molecules are poor binders or have low solubility. Here, we introduce a new soaking method which focuses on single crystals and offers a way to successfully achieve complex formation of macromolecular crystals with potential drug targets.

### 4.1. Ligand and solvent concentration in the crystal   

The AeGe technique uses reservoir-free crystals under a temperature/humidity-controlled gas stream. Target mol­ecules are suspended in an aerosol phase to be delivered onto the crystal surface. In other words, ligands are directly soaked into protein crystals without being diluted in the crystallization drop.

Estimation of ligand concentration in the crystal using DMSO solutions during the spray process is a requirement to gain control of crystal complex formation. Moreover, the amount of dissolved ligand in the crystal correlates with the concentration of solvent in the crystal, which should be as high as possible if dealing with rather insoluble compounds. The ligand concentration of the aerosol solution determines the experimental time to reach a desired final ligand concentration in the crystal and occupy all binding sites.

Regardless of the low n*M* binding affinity of 1G244 for DPP8/9, the low solubility of the compound in aqueous solution might explain the lack of complex formation with DPP8. In order to attain recognizable difference electron density, the proportion of liganded protein should be significant. This is dependent on the incubation time and binding affinity constant (Ki) of the ligand. To achieve more than 90% of protein–ligand complex the ligand concentration should at least ten times greater than the Ki (Müller, 2017 ▸). The use of the AeGe soak allowed us to reach sufficient dissolved ligand molecules in the crystal that this condition was satisfied.

### 4.2. Solvent tolerance of protein crystals   

One strategy to soak compounds with low water solubility is to pre-equilibrate a protein crystal in DMSO, where ligands are soluble. Our experience shows that reservoir-free crystals tolerate more DMSO, perhaps by restraining phase changes from solid to liquid in the absence of mother liquor (Shenoy *et al.*, 2001 ▸; Chernov, 2003 ▸). Even partial covering of the crystal during DMSO spraying does not significantly damage the crystal structure. In classical soaks, crystals in mother liquor containing DMSO seem to be prone to degradation. The gradual increase of DMSO in the crystal accomplished by a humidity gradient while monitoring the crystal volume appears to be a milder method. Importantly, nearly all bulk water in the crystals can be exchanged for DMSO. Moreover, at higher DMSO concentration (>20%) the crystal is ready for cryo-cooling. Volatile solvents have to be supplied continuously to the crystal to replace the evaporated fraction. Otherwise, the crystal volume would decrease, most likely damaging the crystal order. In the case of water as solvent, the humidified gas stream suffices to keep the volume of the crystal constant. In the case of crystal intolerance to DMSO, other solvents like di­methyl­formamide, *N*,*N*′-di­methyl­propyl­eneurea, ethanol, iso­propanol, PEGs, 1,3-di­methyl-2-imidazolidinon and *N*-methyl-2-pyrrolidone are alternatives compatible with the AeGe.

### 4.3. Crystal temperature   

The crystal temperature defined by the humidified gas stream has a significant influence on crystal stability. Crystals tolerate DMSO treatments better at lower temperature (here 12.5°C) than at a standard 19°C. Lower temperature also reduces the evaporation rate of DMSO, reducing mechanical stress for the crystal during the spraying process. However, the use of low gas temperature is limited by the freezing point of the crystal solution. In the case of DMSO the freezing point can reach 18°C. We note that a 100% DMSO aerosol solution remains liquid on the crystal surface even at 12.5°C crystal temperature. On the other hand, the solubility of the solute increases with temperature.

### 4.4. Optical crystal control   

Tracking crystal area changes by optical measurement during the experiment was crucial for a successful structure determination. The crystal volume can be used to identify sudden changes in response to ligands, which might be linked to crystal order. Here, we noticed that at a certain DMSO concentration the crystal started to shrink, although it remained stable and did not dissolve. Upon further supply of DMSO plus ligand, the crystal started to melt, and then entered a collapse phase (dissolution). Before reaching the collapse phase, the crystal was cryo-cooled by the CryoSwitch. This crystal behavior was reproducible upon DMSO treatment. Remarkably, larger crystals tended to be more stable. Therefore, we used only crystals bigger than 100 µm.

### 4.5. Comparison of PD and AeGe   

The PD device was developed to supply picolitre-sized drops to freely mounted crystals. This device has been intensively used for diffraction improvement and ligand complex formation on protein crystals. Nevertheless, it has some disadvantages compared with the AeGe. In Table 2 ▸ we summarize some of the advantages and disadvantages of each system.

The main advantage of the AeGe is a stable delivery of smaller drops to the crystal. The crystal is impinged upon by several picolitre drops distributed over the crystal surface, which implies a reduction of mechanical stress compared with a single larger drop from the PD. The distance required for the PD to reach the crystal with a drop is restricted to a few millimetres. Therefore, the very front tip of the PD is exposed to humidity coming from the humidified air stream, resulting in ligand precipitation which can block the device. In contrast, the aerosol generation is far from the humid air stream and ligand precipitation before aerosol generation is not a problem. Another difference concerns the procedure of loading with solution. The PD has to be dismounted and therefore alignment is necessary for every new experiment. Conversely, a capillary loads the AeGe with solution, which operates independently from the AeGe core unit. The position of the AeGe is fixed once it has been aligned. One major advantage of the AeGe is the compatibility with different solutions. Nearly any type of solution can be used to produce aerosol under similar settings, which is not the case for the PD. The aerosol average drop size increases with the viscosity of the aerosol solution. This is counteracted by operating the AeGe at a frequency beyond 250 kHz, which leads to a reduction of drop size.

### 4.6. Crystallographic structures of DPP8 complexed with 1G244 and EIL peptide   

Two protein model systems confirm the advantages of using the AeGe method in challenging complex formation scenarios. Most strikingly, the AeGe allowed us to generate previously unattainable complexes of 1G244 and the EIL peptide with DPP8 crystals. 1G244 is a model for a water-insoluble mol­ecule and the EIL peptide is a model for a low-affinity binder: neither forms complexes with DPP8 using classical soaking. Moreover, DPP8 crystals are stable only at 4°C. Transferring crystallization plates to 20°C induces immediate instability and in many cases dissolution of crystals. This additional constraint restricts classical soaks to 4°C, rendering even lower compound solubility. However, if crystals are taken out of the drops, they have increased tolerance to temperature and ligands.

Both DPP81G244 and DPP8–EIL peptide structures are in agreement with previous reports (Ross *et al.*, 2018 ▸), as they exhibit a disorder/order transition of an active-site-related protein segment upon inhibitor binding. The 1G244 molecule is fully visible. Importantly, the atoms of the fluoro­benzhydryl substituent occupy S2, but also overlap with S1′ and S2′. The EIL peptide binding mode is very similar to that of the published SLRLFLYEG peptide (Pilla *et al.*, 2013 ▸). However, the reason why the EIL peptide has lower binding affinity compared with SLRFLYEG remains elusive. In fact, the only sequence difference (F to Y) does not provide further clues, since both residues are exposed to solvent without forming strong contacts. Although exosites are expected to play a role at defining specificity, we are unable to identify those sites in this particular structure.

### 4.7. Outlook   

In the future we plan to establish a method upgrade to determine crystal volume instead of projection area. This tool will allow us to calculate the DMSO concentration in the crystal by quantifying a correlation between crystal-volume change relative to humidity change in a dehydration control experiment. Furthermore, the increment of ligand concentration in the crystal can be quickly computed by following the volume of ligand solution delivered to the crystal over time.

## 5. Conclusion   

This report describes a dilution-free soaking method of reservoir-free crystals to bind compounds dissolved in both organic and water solvents. The results suggest that issues of solubility, affinity and crystal deterioration can be overcome by carefully adjusting ligand and solvent concentration in protein crystals using the AeGe.

## Supplementary Material

DPP8_EIL coordinates. DOI: 10.1107/S1600576721003551/gj5259sup1.txt

DPP8_EIL structure_factors. DOI: 10.1107/S1600576721003551/gj5259sup2.txt

DPP8_1G244 coordinates. DOI: 10.1107/S1600576721003551/gj5259sup3.txt

DPP8_1G244 structure_factors. DOI: 10.1107/S1600576721003551/gj5259sup4.txt

Crystal structure of DPP8 in complex with staurosporine soaked by AeGe. DOI: 10.1107/S1600576721003551/gj5259sup5.pdf

PDB reference: DPP8 in complex with the EIL peptide (SLRFLFEGQRIADNH), 6trw

PDB reference: DPP8 in complex with 1G244, 6trx

PDB reference: aerosol-soaked human cdk2 crystals with staurosporine, 7nvq

## Figures and Tables

**Figure 3:**
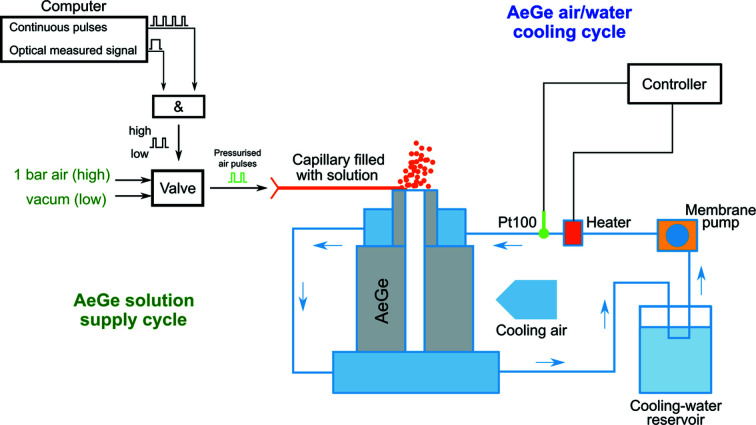
Scheme of the AeGe cooling and liquid supply cycles. Left: solution supply is regulated by defined pressure pulses in time, produced by the digital ‘AND’ wiring of continuous pulses with the signal according to the optical measurement of crystal plus solution (Kiefersauer *et al.*, 2014 ▸). The AeGe is constantly operating during the experiment and the deposited solution is fully nebulized instantaneously. Right: temperature regulation is achieved by cooling parts resting on the top and bottom of the AeGe, which are connected to a cooling circuit, a heater and a temperature sensor. Additional air-cooling is directed to the AeGe.

**Table 1:** Crystallographic parameters for DPP8–1G244 and DPP8–SLRFLFEGQRIADNH liganded structures Unless stated otherwise, values in parentheses correspond to the highest-resolution shell. The cell dimensions for a non-soaked *C*222_1_ crystal are 161.2, 252.2, 261.2 Å, 0.4% greater than those after treatment.

	DPP8–1G244	DPP8–SLRFLFEGQRIADNH
PDB code	6trx	6trw

Data collection		
Space group	*C*222_1_	*C*222_1_
Resolution (Å)	44.38–3.20 (3.28–3.20)	49.16–3.00 (3.08–3.00)

Cell dimensions		
*a*, *b*, *c* (Å)	163.96, 246.29, 261.80	163.00, 245.26, 261.42
α, β, γ (°)	90, 90, 90	90, 90, 90
*R*_meas_ (%)	15.6 (150)	9.7 (135.1)
CC_1/2_ (%)	99.8 (79.9)	99.9 (71.0)
*I*/σ(*I*)	14.51 (2.05)	20.41 (1.99)
Completeness (%)	99.9 (100.0)	99.9 (100.0)
Redundancy	8.33 (8.56)	8.42 (8.52)
Mosaicity (°)	0.232†	0.127†
Total observations	726 516	880 465
Total unique observations	87 206	104 482

Refinement		
*R*_cryst_/*R*_free_ (%)	19.67/23.79	19.30/22.63
No. of reflections	82 845 (4361)‡	99 257 (5225)‡
RMSD bond length (Å)	0.007	0.007
RMSD bond angle (°)	1.184	1.133
No. of atoms	20 800	20 730
Average *B* factor (Å^2^)	99.14	91.04

Ramachandran plot (%)		
Preferred region	94.10	94.75
Allowed region	5.11	4.45

† Mosaicity values are similar to those observed in unbound crystals (0.141°).

‡ Values in parentheses correspond to free *R* value test set (5%).

**Table 2:** Comparison of technical features between the PD and the AeGe (see also text)

Feature	PD	AeGe
Drop diameter (H_2_O)	20–30 µm	7–15 µm
Drop diameter (DMSO)	30–40 µm	20–30 µm
No. of drops per impulse	Single	Multiple
Drop distribution on crystal	Local	Statistical
Crystal targeting	Direct	Indirect
Minimum operation volume	30 µl	10 µl
Stability and reproducibility	Medium	High
Solution compatibility	Medium	High
Device cooling	No	Yes
