# Supplementary material for: Aerosol-based ligand soaking of reservoir-free protein crystals
Source: J Appl Crystallogr. 2021 May 28;54(Pt 3):895–902. doi: 10.1107/S1600576721003551 (PMC8202026; doi:10.1107/S1600576721003551)
Supplement: Supplementary file 5 [file j-54-00895-sup5.pdf]

JOURNAL OF
APPLIED
CRYSTALLOGRAPHY

Volume 54 (2021)

Supporting information for article:

Aerosol-based Ligand Soaking of Reservoir-free Protein Crystals

Breyan Ross, Stephan Krapp, Ruth Geiss-Friedlander, Walter Littmann, Robert Huber and Reiner Kiefersauer

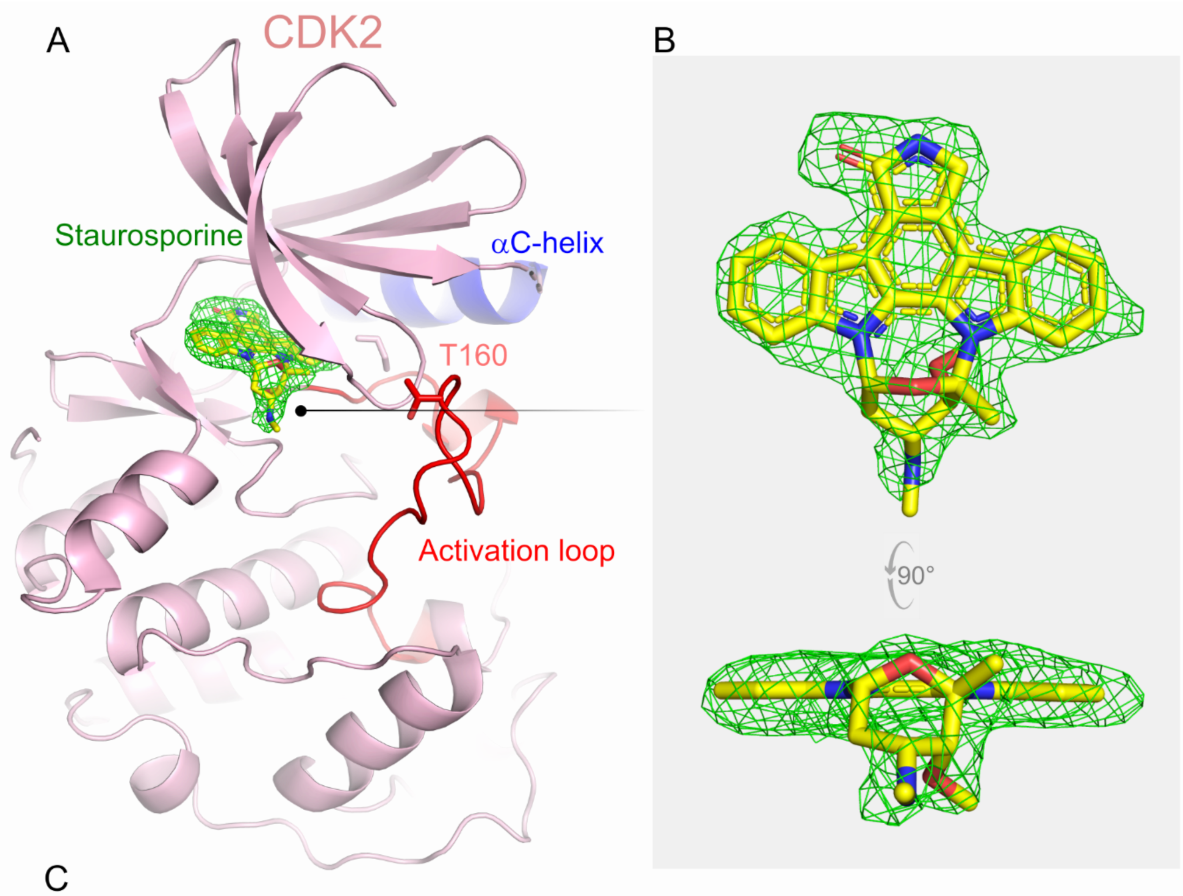

C

**Data collection**		**Refinement**	
Space group	$P2_12_12_1$	R_{cryst}/R_{free} (%)	24.0/28.8
Resolution (Å)	74.42 - 2.05	Number of reflections	14329
**Cell dimensions**		RMSD bond lenght (Å)	0.006
a, b, c (Å)	53.8 69.4 74.4	RMSD bond angles (°)	1.419
α, β, γ (°)	90 90 90	Number of atoms	2400
R_{meas} (%)	14 (100)	Average B-factor ($Å^2$)	34.15
$CC_{1/2}$ (%)	99.3 (83.9)	**Ramachandran plot**	
$I/\sigma(I)$	6.8 (2.3)	Preferred region	273 (96%)
Completeness (%)	87.1 (75.1)	Allowed region	10 (4%)
Multiplicity	6.4 (6.4)	Outliers	0
Mosaicity (°)	2.12		
Total observations	99515		
Total unique observations	15604		

Values in parentheses correspond to the highest-resolution shell

Supplementary figure 1. AeGe soaked crystal of CDK2. The experiment was performed starting with a gas stream humidity of 97% at a temperature of 17°C. A humidity gradient was applied down to 80% in 0.2% steps every 12 s. The aerosol solution contains 10 mM staurosporine dissolved in 100% DMSO and sprayed keeping the crystal volume constant. Only one crystal was needed to finish the experiment and accomplish complex formation **(A)** Human CDK2 monomer complexed with staurosporine. Positive electron density is displayed around the ligand atoms (Fo-Fc - 3σ). **(B)** Zoomed view of staurosporine and its electron density (omit map) in orthogonal view. **(C)** Crystallographic table summarizing data collection parameters and refinement. PDB ID: 7NVQ
